# An improved multiplex RT–quantitative PCR assay can reveal sex-specific activity of transmission-blocking drugs on *ex vivo* gametocytes from *Plasmodium falciparum* asymptomatic infections

**DOI:** 10.1093/jac/dkaf146

**Published:** 2025-05-23

**Authors:** Mariagrazia Ciardo, Noëlie B Henry, Issiaka Soulama, Samuel S Sermé, Dante Rotili, Antonello Mai, Fabrizio Lombardo, Pietro Alano, Giulia Siciliano

**Affiliations:** Dipartimento Malattie Infettive, Istituto Superiore di Sanità, Viale Regina Elena 299, Rome 00161, Italy; Centre National de Recherche et de Formation sur le Paludisme, Ouagadougou, Burkina Faso; Centre National de Recherche et de Formation sur le Paludisme, Ouagadougou, Burkina Faso; Centre National de Recherche et de Formation sur le Paludisme, Ouagadougou, Burkina Faso; Dipartimento di Chimica e Tecnologie del Farmaco, Università degli Studi di Roma La Sapienza, P.le A. Moro 5, Rome 00185, Italy; Dipartimento di Chimica e Tecnologie del Farmaco, Università degli Studi di Roma La Sapienza, P.le A. Moro 5, Rome 00185, Italy; Dipartimento di Sanità Pubblica e Malattie Infettive, Divisione di Parassitologia, Università degli Studi di Roma La Sapienza, P.le A. Moro 5, Rome 00185, Italy; Dipartimento Malattie Infettive, Istituto Superiore di Sanità, Viale Regina Elena 299, Rome 00161, Italy; Dipartimento Malattie Infettive, Istituto Superiore di Sanità, Viale Regina Elena 299, Rome 00161, Italy

## Abstract

**Objectives:**

To develop a multiplex RT–quantitative PCR (RT–qPCR) assay to quantify sex-specific *Plasmodium falciparum* gametocyte transcripts (*pfCCp4*, *pfMGET*), for evaluating the impact of drug treatments on gametocyte viability for malaria transmission-blocking drug development.

**Methods:**

We optimized an RT–qPCR assay incorporating a normalization transcript to use the ΔΔCt method (differences in Cycle threshold) to quantify gametocyte transcript levels. The assay was used on *ex vivo* gametocytes from *P. falciparum* natural infections exposed for 24 h to six drugs impairing mosquito transmission, as measured by the direct membrane feeding assay. Follow-up *in vitro* experiments showed that an additional 48 h incubation, following drug wash-out, was required to monitor decline in transcript levels and potential sex-specific effects.

**Results:**

The optimized assay revealed efficacy of drug treatment as a reduction in transcript levels for two of the six drugs tested: 30% for *pfMGET* and 80% for *pfCCp4* in methylene blue (5 µM)–treated samples, and 75% for both sex-specific transcripts in samples treated with P218 (0.25 µM). In the remaining drugs, a 48 h incubation period post drug wash-out was required to measure a decline in transcript levels. Furthermore, a differential reduction in the levels of male versus female gametocyte transcripts suggested sex-specific effects for two of the drugs.

**Conclusions:**

The multiplex RT–qPCR assay provides a reliable method to assess the inhibitory effects of drug treatments on *P. falciparum* gametocytes, with the potential to evaluate both overall and sex-specific impacts on gametocyte viability. This assay represents a valuable tool in the development and evaluation of transmission-blocking drugs, particularly in distinguishing effects on male and female gametocytes.

## Introduction

The life cycle of *Plasmodium falciparum,* the most dangerous parasite responsible for malaria, involves two hosts: female mosquitoes of the genus *Anopheles* and humans. The transmission of the parasite from humans to mosquitoes hinges on the presence in peripheral blood of gametocytes, the parasite sexual stages. For transmission to succeed, both mature male and female gametocytes must be taken up by a female *Anopheles* mosquito, as the first step to onward transmission is the fertilization of one female gamete by one male gamete.^1^

Key factors influencing transmission efficiency are the gametocyte density and their sex ratio, defined as the proportion of male gametocytes. Over the years, various models have been developed to predict how gametocyte sex ratios impact transmission to mosquitoes.^2,3^ One such model suggests that the density of female gametocytes is a primary predictor of infectivity, with increasing transmission observed at high densities.^4^ Male gametocyte density also affects transmission, particularly at low densities of female gametocytes, where it can limit infection success.^4^ This highlights the importance of accurately measuring both male and female gametocyte densities and their sex ratio in order to potentially predict infectiousness. Additionally, some studies suggest that antimalarial drugs may exert distinct effects on male and female gametocytes.^5,6^ These differences must be considered when developing antimalarial strategies to interrupt transmission.

In the determination of *P. falciparum* gametocyte densities, molecular techniques have emerged as essential tools because very low, submicroscopic gametocytaemias can still efficiently infect mosquitoes.^7–10^ Amongst the messenger RNAs (mRNAs) uniquely expressed in the sexual stages and used in RT–quantitative PCR (RT–qPCR), a few have been used to measure density of female and male gametocytes as they are predominantly expressed only in one sex.^11,12^ One of these is a multiplex RT–qPCR assay that simultaneously amplifies the *pfCCp4* (PF3D7_0903800) and the *pfMGET* (PF3D7_1469900) mRNAs as female- and male-specific markers, respectively,^13^ and has been used to investigate possible sex-specific effects of drug treatments in clinical trials.^12,14^

The development of interventions that block parasite transmission, key for malaria elimination, actively explores the use of anti-gametocyte drugs. Although artemisinin combination therapy is effective against pathogenic asexual stages and immature gametocytes, it does not affect the circulating mature sexual stages, whose residual presence in the bloodstream poses a significant risk of further transmission.^15^ Both for the development of novel drugs and for measuring efficacy of specific drug treatments blocking gametocyte transmission, reliable tools are essential for assessing the effectiveness of any transmission-blocking drugs. Presently, no reliable marker or molecular assay, truly predictive of transmission-blocking efficacy of drug treatments, is available to complement complex, long, costly, low-throughput and potentially hazardous assays like the standard membrane feeding assay (SMFA) or direct membrane feeding assay (DMFA), which remains the gold standard.^16–18^

A further limitation in testing compounds or drug candidates for transmission-blocking activity is the use of *P. falciparum* lines established in laboratory settings, which can miss insights into the effects of drugs on gametocytes in the human body and/or on wild gametocytes in natural infections. Recent advances have addressed this with SMFA using gametocytes sampled from drug-treated human volunteers to assess drug transmission-blocking activity,^12,14^ and protocols to obtain wild gametocytes from naturally infected individuals and to maintain them in *ex vivo* cultures for variable times from a few minutes to 48 h, when they can be exposed to drugs and compounds prior to testing infectiousness in SMFA.^19–21^

The present work used an improved RT–qPCR multiplex assay to measure gametocyte sex-specific transcripts on one of the above set of gametocytes from *P. falciparum* natural infections.^21^ In that study, *ex vivo* gametocytes from naturally infected blood samples were exposed for 24 h to a panel of drugs and compounds and were subsequently tested by DMFA for the ability to transmit the parasite in *Anopheles* mosquitoes. Here, we measured the impact of drug treatments that caused a block in parasite transmission on the level of two sex-specific transcripts with the aim to investigate if the assay can be used to measure gametocyte fitness and infectiousness to mosquitoes, and to detect possible sex-specific effects of drug treatments.

## Materials and methods

### Description of the ex vivo gametocyte samples

Details of the collection of blood samples from 11 individuals naturally infected with *P. falciparum* and of the drug treatment of the *ex vivo* gametocytes are contained in Henry *et al.*^21^ and in Table S1 (available as Supplementary data at *JAC* Online). Parallel aliquots of treated and control blood samples were centrifuged and resuspended in RNAlater solution (Invitrogen) and stored at −80°C until RNA extraction. In this experiment only compounds that had shown transmission-blocking activity in mosquitoes in the DMFA were included.

### Parasite culture and gametocyte production

The *P. falciparum* NF54 line^22^ was cultured in human O^+^ erythrocytes according to the standard method.^23^ To induce gametocyte production for the *in vitro* experiments, a culture at 10%–13% parasitaemia was treated with 0.5 M *N*-acetylglucosamine (NAG; Sigma A3286) to remove asexual parasites. Stage II–III gametocytes were purified using a modified Percoll 60% gradient^24^ and allowed to develop to stage V. After counting in a Neubauer chamber, 2.5 × 10^5^ stage V gametocytes were used per sample in 50 µL of whole blood. Control and drug-treated gametocyte samples were suspended in 10 volumes of TRIzol^™^ reagent (Invitrogen) and stored at −80°C until RNA extraction. Three biological replicates were performed for each treatment at each incubation time (see Table S2).

### Antimalarial compounds

Drugs and compounds used, as detailed in Henry *et al*.,^25^ were stored in DMSO as stock solutions at 10 mM at −20°C. The synthesis of compound MMV085203 is described in the Supplementary material. The final compound was stored in DMSO as stock solutions at 10 mM at −20°C.

### RNA extraction

The RNAlater^™^ stabilization solution was removed from the *ex vivo* gametocyte samples by centrifugation at 5000 **g** and the pellet was resuspended in 10 volumes of TRIzol^™^. For both the *ex vivo* and the *in vitro* gametocyte samples, 0.2 mL of chloroform (Sigma-Aldrich, 34854) was added per 1 mL of TRIzol^™^, mixed for 2–3 min and centrifuged for 15 min at 12 000 **g** at 4°C to separate the RNA-containing aqueous phase. RNA was extracted from 50 µL in the MagPurix^®^ 12 EVO (Zinexts Life Science Corp.) with the MagPurix^®^ Total RNA Extraction Kit (ZP02019), eluted in 50 µL and quantified with the NanoPhotometer NP80-Implen.

### RT–qPCR protocol

Primers and probes are shown in Table 1. Human *GAPDH* sequences are from the TaqMan^™^  *GAPDH* Assay with JUN^™^ dye/QSY^™^ probe (cat. number: 4485712) from Applied Biosystems^™^. The RT–qPCR reactions, combining reverse transcription and PCR, were performed using 5 µL of RNA in a final reaction volume of 20 µL, in triplicate, with the TaqPath^™^ 1-Step Multiplex Master Mix (No ROX) from Applied Biosystems, using the QuantStudio 5 real-time PCR system (Thermo Fisher Scientific) with the following programme: 55°C 15 min, 95°C 1 min, 44 cycles at 95°C 10 s and 60°C 1 min. QuantStudio Design & Analysis Software was used to analyse data.

**Table 1.** Primer and probe sequences for RT–qPCR assays

Target gene	Primer/probe	Sequence (5′→3′)	Reference
*pfMGET* (male-specific)	Forward primer	ATGCCATGTTGATGTTGTCCTG	Meerstein-Kessel L *et al.*, 2018^13^
*pfMGET* (male-specific)	Reverse primer	TGTTGATGTTGGTGGTGGTGTA	Meerstein-Kessel L *et al.*, 2018^13^
*pfMGET* (male-specific)	Probe	6-FAM-CGTTGCTGGTGCTGTGGTG-BHQ1	Meerstein-Kessel L *et al.*, 2018^13^
*pfCCp4* (female-specific)	Forward primer	AGATGCCGACGAGTTCACCT	Meerstein-Kessel L *et al.*, 2018^13^
*pfCCp4* (female-specific	Reverse primer	CCATGCCGACTTCCTTCTTGT	Meerstein-Kessel L *et al.*, 2018^13^
*pfCCp4* (female-specific)	Probe	6-FAM-CGTCCTGTGATCCTGCCTGC-BHQ1	Meerstein-Kessel L *et al.*, 2018^13^
*GAPDH* (human reference)	Forward primer	Provided in TaqMan^™^ *GAPDH a*ssay	Applied Biosystems^™^
*GAPDH* (human reference)	Reverse primer	Provided in TaqMan^™^ *GAPDH* assay	Applied Biosystems^™^
*GAPDH* (human reference)	Probe	JUN^™^ dye/QSY^™^ probe (cat. number: 4485712)	Applied Biosystems^™^

### ΔΔct method

The method compares the difference in Cycle threshold (ΔCt) between the transcript of interest (*pfCCp4* or *pfMGET*) and the reference transcript (*GADPH*) in the treated and untreated control samples. Preliminary standard curves were determined by RT–qPCR assays on serial dilutions of RNA from gametocyte-infected blood samples (see Supplementary material, Figure S1), confirming similar amplification efficiency across all genes. The ΔCt is calculated as the difference between the target gene and *GAPDH* Ct values. The ΔΔCt is then computed by subtracting the ΔCt of the DMSO control from the treated sample, and relative gene expression changes are calculated using the formula 2^−ΔΔCt^, assuming 100% amplification efficiency.

### Graphs and statistical analysis

Statistical analyses were performed using GraphPad Prism (version 6.01) and Microsoft Excel (version 2016). *P* values were calculated using *t*-tests with a 95% CI and a two-tailed distribution. The significance threshold was set at P = 0.05. The *t*-test was selected since the comparisons in our study were independent, and the data for gametocyte transcript levels (Ct values) followed a normal distribution. This made the *t*-test an appropriate choice for comparing means between the two groups.

## Results

### Improved multiplex RT–qPCR assay for gametocyte sex-specific markers to assess effects of drug treatment on ex vivo gametocytes of P. falciparum

We analysed by RT-qPCR *P. falciparum ex vivo* gametocyte samples treated in parallel with drugs and compounds that successfully blocked parasite transmission, as described in Henry *et al.*^21^ Six drugs/compounds were incubated, some in two concentrations, on three to four aliquots of gametocyte-infected blood from a total of 11 donors (Table S1).

The aim of this analysis was to evaluate the effect of drug treatment on the levels of the transcripts *pfCCp4* and *pfMGET*, markers of female and male gametocytes, respectively. In order to use the ΔΔCt method for this analysis,^26^ we modified the dual RT–qPCR assay described in Meerstein-Kessel *et al.*^13^ by introducing a third marker, the human housekeeping gene *GAPDH*, for normalization of total RNA quantity. Quality control data for use of the three amplicons in this multiplex assay are shown in the Supplementary material, and use of the ΔΔCt method on these samples is detailed in ‘Materials and methods’. In brief, this method compares the difference between the *GAPDH*-normalized amounts of the target mRNAs (*pfCCp4* or *pfMGET*) in pairs of drug-treated and DMSO control samples. The difference is expressed by the value 2^−ΔΔCt^: values of 2^−ΔΔCt^ close to 1 indicate that levels of the specific transcripts are not affected by drug treatment, whereas values <1 reveal that the treatment led to a decrease in mRNA levels. The method also yielded values of 2^−ΔΔCt^ > 1, suggesting increased levels of mRNA after treatment; however, we decided to overlook such possibly interesting cases in the absence of specific follow-up experiments, as the focus of this analysis was to test if potential reductions in transcript levels after treatment can be used as proxies of drug-induced gametocyte inhibition/death.

**Figure 1.**
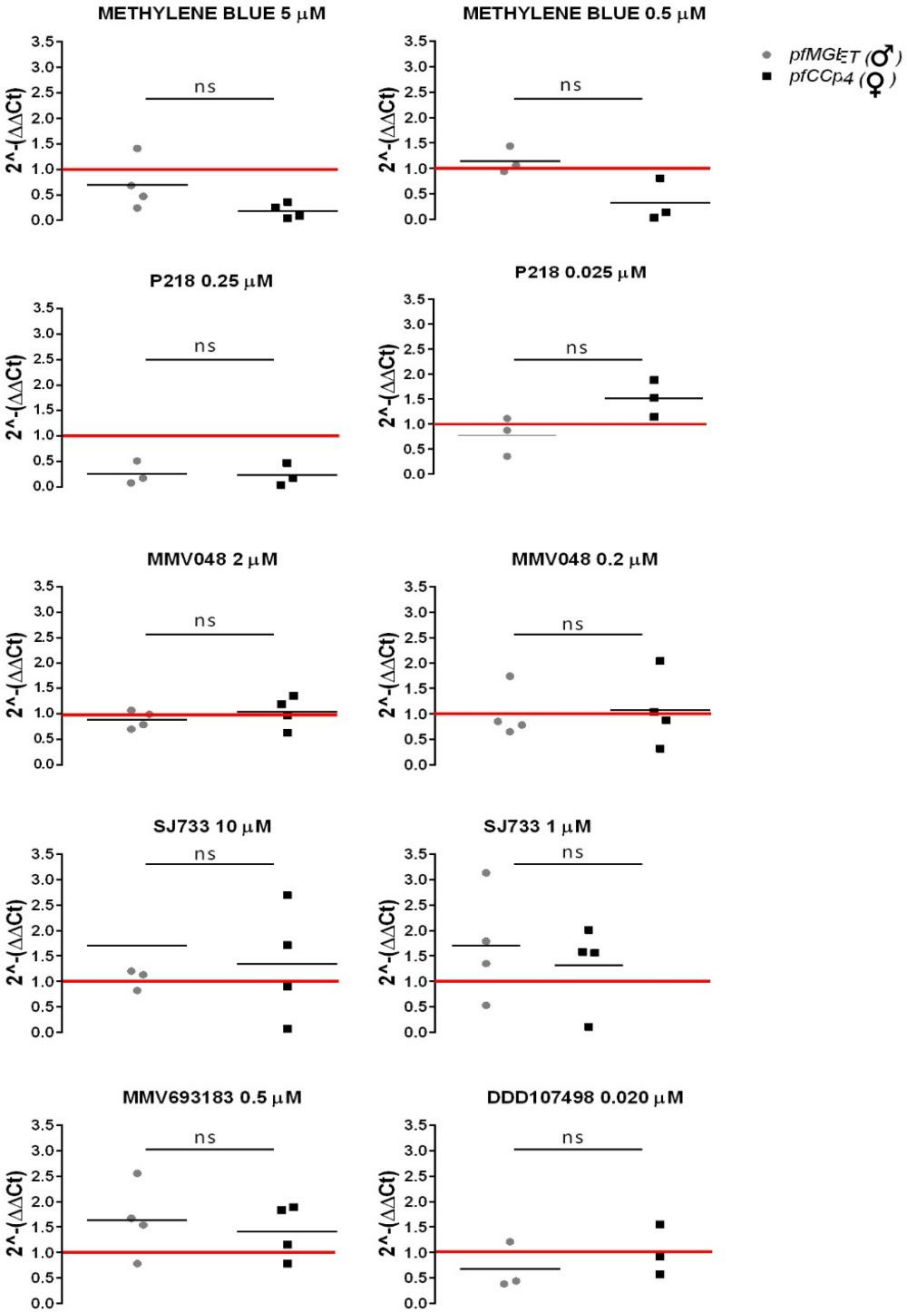
ΔΔCt analysis of *pfCCp4* and *pfMGET* transcript levels in *ex vivo* gametocytes following a 24 h drug/compound treatment. The plots indicate the effect of the compounds on the expression level of the gametocyte sex-specific targets *pfMGET* and *pfCCp4* expressed as 2^−ΔΔCt^ values. The 2^−ΔΔCt^ value = 1 (red line) indicates that the treatment does not affect transcript levels. ns, not significant,  *P* value >0.05, 95% CI indicates that treatment does not result in a significant difference in the relative levels of the sex-specific markers.

Results of this analysis are shown in Figure 1. Treatment with the higher doses of methylene blue (MB) 5 µM and P218 0.25 µM resulted in the decrease of both sex-specific mRNAs. A decrease in the level of both markers was not measurable at 10-fold lower doses of these drugs, although the 2^−ΔΔCt^ values for the female marker *pfCCp4* were <1 in all samples treated with MB 0.5 µM and the 2^−ΔΔCt^ values of the male marker *pfMGET* were <1 in two of three samples treated with P218 0.025 µM. Besides these cases, the treatments with the other drugs, namely MMV048 (2 µM and 0.2 µM), SJ733 (10 µM and 1 µM), MMV693183 (0.5 µM) and DDD107498 (0.02 µM) did not cause obvious decreases in transcript levels for either marker.

### Detection of decrease of sex-specific transcripts requires an extended gametocyte cultivation time after drug treatment

As the above drug treatments blocked the ability of the *ex vivo* gametocytes to infect mosquitoes,^21^ the decrease in both gametocyte sex-specific transcripts 24 h after the MB and P218 high-dose treatments was likely diagnostic of the previously described lethality of these drugs for the parasite sexual stages.^27,28^ We hypothesized that the failure to observe a similar decrease in mRNA levels for lower doses of MB and P218 or the other transmission-blocking drugs was due to the fact that 24 h was insufficient to cause a detectable decline in transcript levels in the affected gametocytes. To test this hypothesis, *P. falciparum* NF54 gametocytes cultured *in vitro* (3000 parasites/µL) were treated with the same drugs/compounds used on the *ex vivo* gametocytes for the same 24 h incubation time but, after drug removal, they were further incubated for an additional 48 h. Samples for the RT–qPCR analysis were taken at both the 24 h and the 24 + 48 h timepoints.

**Figure 2.**
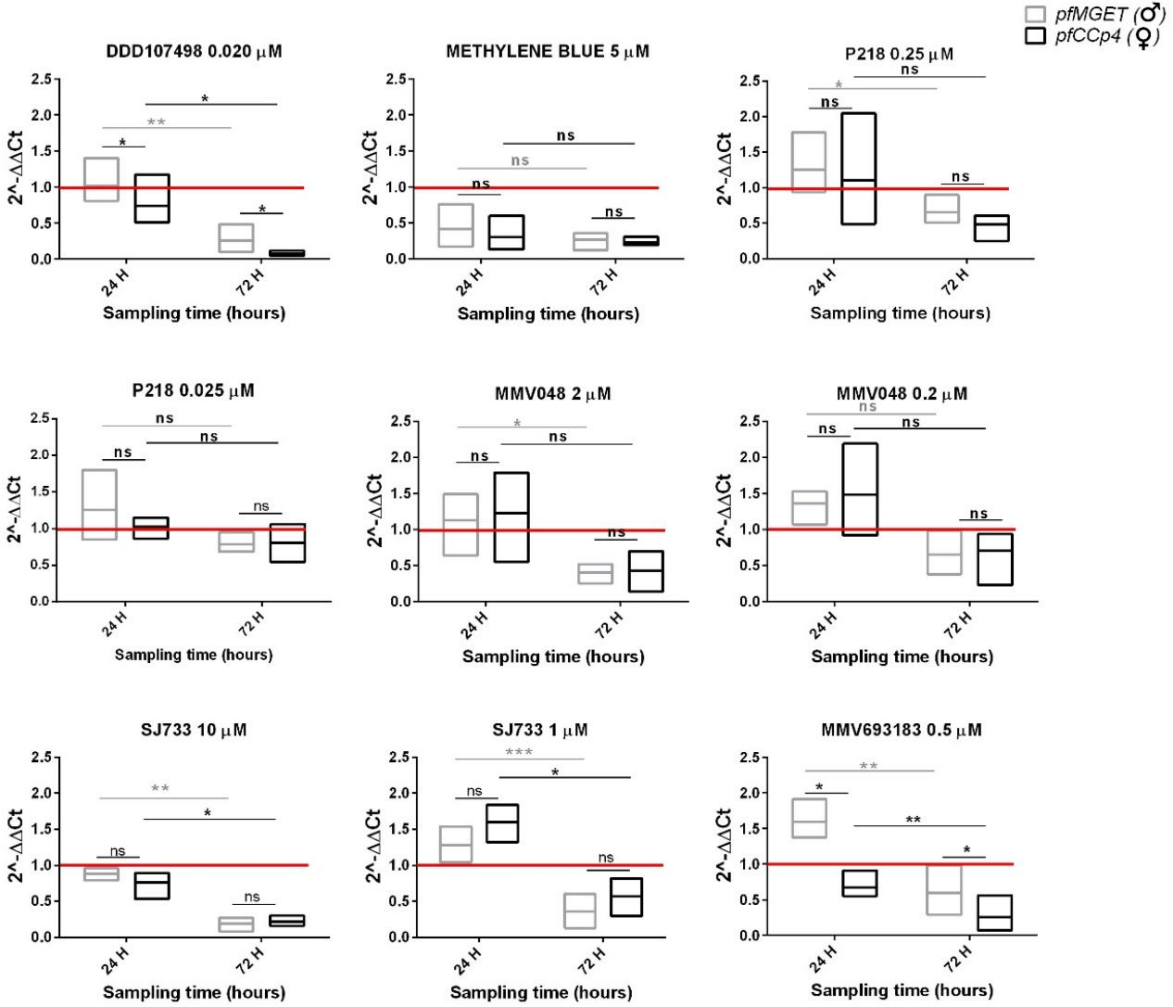
ΔΔCt analysis of *pfCCp4* and *pfMGET* transcript levels in *in vitro* NF54 gametocytes after a 24 h drug/compound treatment followed by an additional 48 h incubation time (72 h sampling time). The ΔΔCt values were obtained for each target at the 24 h and 24 + 48 h timepoints. The 2^−ΔΔCt^ value = 1 (red line) indicates that the treatment does not affect transcript levels. Three biological replicates were performed, each one analysed in a technical triplicate. In box plots, the centre line represents the mean. The red line represents the transcript level of the DMSO control samples, which is set to a value of 1. This serves as the baseline for comparison, where values <1 indicate decreases in transcript levels relative to the control. Effect size: Cohen's *d* values are >2.5, indicating a large effect size for significant comparisons between the 24 h and 72 h timepoints; Cohen's *d* values are >1.2, indicating a moderate effect size for significant comparisons between male and female gametocytes; 95% CIs were calculated for all key comparisons. ns, not significant,  *P* value >0.05; *P value <0.05; **P value <0.01; ***P value <0.001; 95% CI.

Results of this experiment are shown in Figure 2. With the exception of P218 0.025 µM, this analysis showed that indeed a decrease in *pfCCp4* or *pfMGET* transcript levels was readily measurable in the samples after the extended incubation time. In the case of MMV048, SJ733 and P218 the decrease was more marked at the higher concentration. Notably, the decrease in *pfMGET* transcript levels after 24 + 48 h was significant, whereas the decrease in *pfCCp4* was less pronounced for MMV048 2 µM and P218 0.250 µM due to the wide range of the *pfCCp4* 2^−ΔΔCt^ values at the 24 h timepoint.

This result confirmed that an extended drug-free incubation time of 48 h of the treated gametocytes is necessary and, in most cases, is sufficient to detect the mRNA degradation of the sex-specific transcripts in the dead/dying sexual stages.

### Multiplex RT–qPCR assay can reveal sex-specific effects of drug treatment

In addition to diagnosing gametocyte killing/inhibition, the RT–qPCR assay was also able to reveal sex-specific effects of drug treatment for two of the compounds under study.

Incubation with MMV693183 at 0.5 µM led to a 2^−ΔΔCt^ value <1 only for the female marker *pfCCp4* after 24 h and a significant decrease (*P* value 0.03) of *pfCCp4* compared with *pfMGET* at the extended incubation time (Figure 2, bottom right panel). Interestingly, this compound showed no effect on either transcript of the *ex vivo* gametocytes. Similarly, treatment with DD107498 at 0.020 µM caused a significant decrease (*P* value 0.05) in the level of the female *pfCCp4* mRNA at the extended incubation time (Figure 2, top left panel).

**Figure 3.**
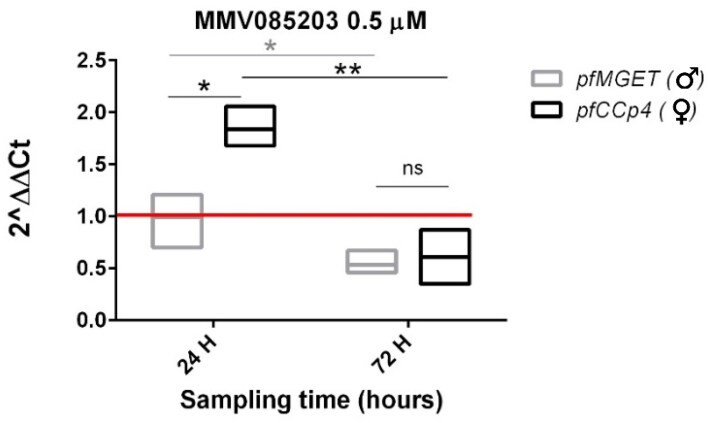
ΔΔCt analysis of *pfCCp4* and *pfMGET* transcript levels in *in vitro* NF54 gametocytes after a 24 h MMV085203 treatment and an additional 48 h incubation time (72 h sampling time). RT–qPCR analysis showed no significant reduction in either *pfCCp4* or *pfMGET* transcript levels. Extending the observation period to 72 h revealed a reduction of over 40% in both *pfCCp4* and *pfMGET* transcripts. The 2^−ΔΔCt^ value = 1 (red line) indicates that the treatment does not affect transcript levels. Three biological replicates were performed, each one analysed in a technical triplicate. The red line represents the transcript level of the DMSO control samples. In box plots, the centre line represents the mean. Effect size: Cohen's *d* values are >2.5, indicating a large effect size for significant comparisons between the 24 h and 72 h timepoints; Cohen's *d* values are >1.2, indicating a moderate effect size for significant comparisons between male and female gametocytes; 95% CIs were calculated for all key comparisons. ns, not significant,  *P* value >0.05; *P value <0.05; **P value <0.01; ***P value <0.001; 95% CI.

To test whether our assay could also detect cases in which drug treatments led to a differential decrease of the male marker *pfMGET*, we performed the same analysis on NF54 gametocytes treated with compound MMV085203. This molecule has been previously reported to exhibit a male gametocyte–specific inhibition in the dual gamete formation assay (DGFA) and transmission-blocking activity in the SMFA. The latter assay was conducted after compound wash-out, which indicated that the transmission-blocking effects were entirely due to pre-feeding gametocyte inactivation.^6^ Our experiment showed that the MMV085203 0.5 µM treatment produced a decrease in transcript levels, similar for both the male- and the female-specific markers, detectable only after the extended incubation time (Figure 3). After 24 h the 2^−ΔΔCt^ value was >1 for the female *pfCCp4* transcript compared with the 2^−ΔΔCt^ value of 1 for *pfMGET*, indicating that levels of the male-specific transcript were not affected by MMV085203.

## Discussion

In the current study, the multiplex RT–qPCR assay to measure male- and female-specific gametocyte transcripts developed by Meerstein-Kessel *et al.*^13^ was modified, and implemented to investigate whether changes in transcript levels after treatment with a panel of six transmission-blocking drugs could predict gametocyte infectivity in mosquitoes. Samples of *ex vivo* gametocytes from natural *P. falciparum* infections, exposed to a 24 h drug treatment and tested in parallel for infectivity in mosquitoes,^22^ were used in the modified assay to measure levels of the male- and female-specific target mRNAs and data were analysed by the ΔΔCt method.

The robustness of the approach was demonstrated by the consistent Ct values for the *GAPDH* normalization marker, across samples from the same infected individual (Table S1), indicating that RNA extractions had comparable yields and that treatments did not affect amounts of this transcript.

This analysis revealed that 24 h treatments with MB 5 µM and P218 0.25 µM caused a reduction in transcript levels of both sex-specific markers. This result is consistent with the potent gametocytocidal activity of MB, previously described using cell-based assays with luciferase reporters specifically expressed in the transgenic parasite sexual stages,^27,29^ even though morphological integrity of gametocytes was unaffected.^27^ The reduction in transcript levels of *pfCCp4* and *pfMGET* in a 24 h treatment thus indicates that our assay can be used to monitor MB-mediated killing of gametocytes also in non-transgenic gametocytes, *ex vivo* from natural infections or produced by WT laboratory lines.

P218 is a potent antimalarial drug that targets the dihydrofolate reductase (DHFR) enzyme in *P. falciparum*, disrupting folate metabolism and DNA synthesis.^28^ At lower concentrations, P218 demonstrated efficacy in reducing male-specific transcript levels by 20% compared with female-specific transcript levels, whose 2^−ΔΔCt^ value was >1. Albeit non-significant (*P* value 0.1), this reduction may suggest that P218 acts faster on male versus female gametocytes even at the lower dose. To the best of our knowledge, sex-specific effects of this drug have not been investigated, although an inhibitory effect on male gametocyte exflagellation has been reported.^28^

The assay results on the *ex vivo* gametocyte samples, however, showed that lower doses of MB and P218 as well as the treatments with the remaining four drugs were not associated with detectable changes in the mRNA levels of *pfCCp4* and *pfMGET*.

To investigate this further, a follow-up *in vitro* analysis showed that an extended cultivation period of the treated gametocytes is necessary for the assay to detect reductions in the target transcript levels in the affected, non-dividing gametocytes. This observation is consistent with the similar finding that an extended incubation time is required to measure declines in parasite lactate dehydrogenase (pLDH) activity in drug-killed gametocytes in the adaptation to gametocytes of the pLDH enzymatic assay, originally developed for the proliferating asexual stages.^30^ The introduction of the additional 48 h post-treatment incubation allowed us to detect a significant decline in *pfCCp4* and *pfMGET* transcript levels across all the treatments. Notably, P218 appeared more potent against field isolates compared with the NF54 laboratory strain after 24 h of treatment, aligning with observations from Henry *et al.*,^21^ who reported that P218 was the only compound that showed higher efficacy against field gametocytes compared with *in vitro* cultured ones. In contrast, other compounds did not exhibit significant differences in efficacy between field and *in vitro* gametocytes. These findings may suggest that the genetic background of the gametocytes could play a significant role in modulating drug response, either enhancing or reducing susceptibility to specific drug treatments, underlying the need to assess drug efficacy and optimize antimalarial strategies across diverse parasite populations. To mitigate this aspect, here compounds were tested on gametocytes from multiple individuals to evaluate activity across a range of parasite genetic backgrounds.

A notable result of our study was that the modified RT–qPCR assay could detect gametocyte sex-specific effects of compounds DDD107498 and MMV693183. DDD107498 targets the parasite translation elongation factor 2 (eEF2; PF3D7_1451100), and the observation that this protein is more abundantly expressed in female than in male gametocytes^11^ may be consistent with the sex-specific inhibitory activity of DDD107498 suggested by our assay.

MMV693183, a first-in-class acetyl-CoA synthetase inhibitor,^31^ also showed in our assay a stronger inhibition on female gametocytes. This is consistent with previous observations that MMV693183 preferentially inhibits female gametocyte activation, with an IC_50_ of 12 nM compared with the 100-fold higher IC_50_ of 1 µM measured on male gamete formation.^31^

Besides the ability to detect the above female gametocyte preferential activities, we evaluated whether our assay was also able to reveal specific activity of molecules targeting male gametocytes. To this aim, MMV085203, a compound with specific activity against male gametocytes, was tested. This compound, targeting the parasite multidrug resistance modulator PfMFR3 (PF3D7_031250),^32^ was previously characterized using the *P. falciparum* DGFA, which indicated a specific inhibition of male gametocyte exflagellation following a 24 h treatment.^6^ Unexpectedly, our assay revealed a significant reduction in both *pfCCp4* and *pfMGET* transcript levels, only in the post-treatment extended incubation time, suggesting that treatment negatively impacted both male and female gametocytes, a result that is consistent with the fact that the *pfMFR3* transcript is not differentially expressed in the two sexes.^11^ The discrepancy between the results of the phenotypic DGFA and our molecular assay confirms the importance of relying on different assays to define activity and specificity, and eventually drive target characterization, of gametocyte-active compounds and drugs.

The results of this study indicate that measuring transcript abundance after drug treatment may functionally diagnose the death or the unhealthy conditions of the non-dividing gametocytes. This assay can analyse response to drug treatment in WT sexual stages from natural infections, thereby expanding and complementing the information obtained with reporter-based gametocyte assays on transgenic parasite lines.

Given the non-dividing nature of the target cells, an extended cultivation period after compound treatment is needed, as in the case of the gametocyte pLDH assay,^33^ to reveal the decrease in mRNA levels in the affected sexual stages. Here we used *ex vivo* gametocyte samples maintained and drug-treated for 24 h *in vitro* after withdrawal from the infected individuals.^21^ Introduction of the extended incubation period is compatible with recently published protocols in which *ex vivo* gametocytes are maintained and drug treated for as long as 48 h before testing infectiousness to mosquitoes.^20^ Although the introduction of an additional 48 h post-treatment incubation time proved to be feasible here, in field studies^20^ and in medium- and high-throughput gametocyte assays^33^ to detect drops of transcript and enzymatic markers, respectively, it would be valuable to explore and develop alternative markers or approaches requiring shorter incubation times to simplify the assay.

In conclusion, the modified gametocyte sex-specific assay described here provided a reliable readout for the inhibitory effects of drug treatment on *ex vivo* gametocytes, confirmed by the results of the gold standard mosquito infection experiments conducted on the same samples.^21^ The assay could thus contribute to monitoring gametocyte viability and has the potential to predict infectiousness to mosquitoes. The ability to also reveal a differential toxicity of molecules on female or male gametocytes adds value to investigate both the role of sex ratio and the effects of its modification on parasite transmissibility. In the context of drug discovery, this fairly simple and cost-effective assay can complement existing methods, enabling researchers to prioritize compounds for the further step of the resource-demanding SMFA and DMFA, which require specialized equipment and experienced personnel.

## Supplementary Material

dkaf146_Supplementary_Data

## Supplementary data

Tables S1 and S2 and Supplementary material are available as Supplementary data at *JAC* Online.
