# Supplementary material for: An improved multiplex RT–quantitative PCR assay can reveal sex-specific activity of transmission-blocking drugs on ex vivo gametocytes from Plasmodium falciparum asymptomatic infections
Source: J Antimicrob Chemother. 2025 May 23;80(7):1907–14. doi: 10.1093/jac/dkaf146 (PMC12209802; doi:10.1093/jac/dkaf146)
Supplement: dkaf146_Supplementary_Data [file dkaf146_supplementary_data.docx]

# An improved multiplex RTqPCR assay can reveal sex specific activity of transmission blocking drugs on *ex vivo* gametocytes from *Plasmodium falciparum* asymptomatic infections

Mariagrazia CIARDO^1^, Noëlie B. HENRY^2a^, Issiaka SOULAMA^2b^, Samuel S. SERMÉ^2^, Dante ROTILI^3^, Antonello MAI^3^, Fabrizio LOMBARDO^4^, Pietro ALANO^1^#, Giulia SICILIANO^1^#*

Supplementary information

**Supplementary Figure S1.** **Validation of the parameters required to apply the ΔΔCT method**.


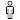


♂

♀

**A**. Amplification curves of the pfCCp4, pfMGET and GAPDH transcripts in serial dilutions of RNA extracted from 1.4 x 10^5^ gametocytes resuspended in whole blood showing the values of the Slope, R^2^ and amplification efficiency for each target. N=3. **B**. ΔCt values (Ct target gene-Ct GAPDH) plotted against the logarithm of the ng of RNA to calculate the slope of the resulting line.

***ΔΔCt validation method***

The use of the ΔΔCt method preliminarly requires that the amplification efficiency of all target sequences, here those of the *pfCCp4*, *pfMGET* and human *GAPDH* transcripts, must be similar and close to 100%. This parameter is calculated by obtaining preliminary standard curves from the respective RTqPCR assays on serial dilutions of RNA extracted from a sample of 1.4 x 10^5^ NF54 gametocytes resuspended in 100 microliters of whole blood. An aliquot of 100 ng of RNA was used to obtain five 1:5 serial dilutions, and each was used to amplify in multiplex mode the gametocyte sex specific transcript *pfCCp4* and *pfMGET* and the human *GAPDH* transcript. From the amplification curves (Figure S1A) the value of the slope, R^2^ and amplification efficiency were calculated. The amplification efficiencies of the three target sequences, ranging between 97 and 112% were therefore adequate to analyse the RTqPCR data with the ΔΔCt method. To confirm similar amplification efficiencies, ΔCt values between the target genes (*pfCCp4* and *pfMGET*) and the reference gene (*GAPDH*) are calculated for each RNA dilution. These ΔCt values are plotted against the log of the ng of RNA, and the slope of the resulting line is measured. A slope close to zero indicates similar amplification efficiencies, validating the use of the ΔΔCt method.

In detail, the following steps are performed: 1) in each dilution, ΔCt values are calculated by subtracting the Ct value of the reference *GAPDH* gene from the ΔCt values of each sex specific target; 2) the resulting *pfCCp4* and *pfMGET* ΔCt values are plotted vs the Log of the RNA ng contained in the diluted RNA samples; 3) the slopes of the lines best fitting the points in the plot are calculated. The slope values close to zero suggest that the amplification efficiencies of the target and reference genes are similar. The slope values obtained range from 0.03 to 0.06 (Figure S1B), therefore indicating that the ΔΔCT method may be used to analyse the data obtained in the modified RTqPCR assay.

***Synthesis of compound MMV085203***

MMV085203 [**1**] was prepared starting from the commercial compound 2,3-dichloronaphthalene-1,4-dione [**2**] through an initial nucleophilic substitution of one chlorine atom with commercially available *tert*-butyl (2-methoxyphenyl) carbamate [**3**] in anhydrous THF using *n*-butyllithium in hexane as base, followed by a second nucleophilic substitution of the remaining chlorine atom by piperidine, employing 1-pentanol as solvent under microwave irradiation. The desired final compound MMV085203 [**1**] was isolated as free base (yield: 68%) after treatment of the Boc protected intermediate **4** with trifluoroacetic acid (TFA) followed by a basic workup, extraction and preparative RP-HPLC purification (Supplementary Figure S2) ^1^ H-NMR (400 MHz, DMSO) δ 8.04 (s, 1H), 7.87-7.95 (m, 2H), 7.71-7.77 (m, 2H), 6.76-6.95 (m, 4H), 3.73 (s, 3H), 2.90-3.05 (m, 4H), 1.22-1.33 (m, 4H), 1.13-1.21 (m, 2H).

**Supplementary Figure S2. Synthesis of the compound MMV085203**


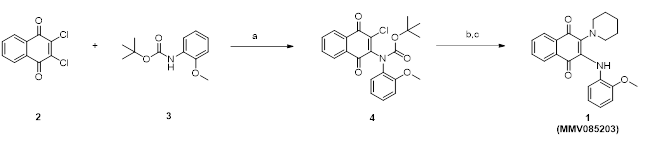


**Reagents and Conditions.** a) Anhydrous THF, n-BuLi in hexane, -78 °C to r.t., 16h; b) piperidine, 1-pentanol, MW, 150 °C, 5 min; c) TFA, rt, 2h then NaHCO_3_.

**Reference**

1. FALOON P, Weiner WS, Smith RA, et al. Inhibitors of the mitf molecular pathway. 2014.

Supplementary Table S1

*Ex vivo* gametocytes

N.T. = Not tested

Supplementary Table S2

*In vitro* gametocytes
